# WebArray: an online platform for microarray data analysis

**DOI:** 10.1186/1471-2105-6-306

**Published:** 2005-12-21

**Authors:** Xiaoqin Xia, Michael McClelland, Yipeng Wang

**Affiliations:** 1 Genomic Core Facility, Sidney Kimmel Cancer Center, San Diego, CA 92121, USA; 2 Department of Cancer Genetics, Sidney Kimmel Cancer Center, San Diego, CA 92121, USA

## Abstract

**Background:**

Many cutting-edge microarray analysis tools and algorithms, including commonly used limma and affy packages in Bioconductor, need sophisticated knowledge of mathematics, statistics and computer skills for implementation. Commercially available software can provide a user-friendly interface at considerable cost. To facilitate the use of these tools for microarray data analysis on an open platform we developed an online microarray data analysis platform, WebArray, for bench biologists to utilize these tools to explore data from single/dual color microarray experiments.

**Results:**

The currently implemented functions were based on limma and affy package from Bioconductor, the spacings LOESS histogram (SPLOSH) method, PCA-assisted normalization method and genome mapping method. WebArray incorporates these packages and provides a user-friendly interface for accessing a wide range of key functions of limma and others, such as spot quality weight, background correction, graphical plotting, normalization, linear modeling, empirical bayes statistical analysis, false discovery rate (FDR) estimation, chromosomal mapping for genome comparison.

**Conclusion:**

WebArray offers a convenient platform for bench biologists to access several cutting-edge microarray data analysis tools. The website is freely available at . It runs on a Linux server with Apache and MySQL.

## Background

Microarray techniques are being used more and more widely, and many models and algorithms have been developed for microarray data analysis. However, in many cases, people need to have a sufficient knowledge of mathematics, statistics and computer skills in order to utilize these methods. A perfect example is Bioconductor [1]. As a leading open source project on genomic data analysis, Bioconductor gathered a wide range of packages available for the analysis of microarray data. However, command line interface programming skills are essential for using Bioconductor and R computer language, which could be an impediment to many biologists. To bridge the gap between biologist's real world problems and the best microarray data analysis methods, we developed WebArray to assist biologists with using tools for microarray data analysis, including some packages from Bioconductor and others.

limma (Linear Models for Microarray Analysis) is one of the most commonly used packages in Bioconductor, which has incorporated the most cutting-edge statistical analysis methods, providing normalization and statistical analysis for cDNA microarray. The key function of the limma package is an implementation of the empirical Bayes linear modeling approach of Smyth [2]. Affy, another commonly used package from Bioconductor, is used for reading Affymetrix GeneChip CEL file, followed by background correction, normalization, probe specific background correction and summarizing the probe set values into one expression measure. Users also have the options of obtaining expression values that correspond to those from the robust multi-array average (RMA) method [3], MAS 5.0 or Li and Wong's MBEI (dchip) [4].

In the context of testing thousands of genes, the false discovery rate (FDR) may be a better way to specify the confidence of microarray. A separate package, spacings LOESS histogram (SPLOSH), which estimates the conditional FDR (cFDR), the expected proportion of false positives conditioned on having k 'significant' findings, has been incorporated into WebArray for further estimating the occurrence of false positives, false negatives and the FDR [5].

Chromosome location mapping is not only important in comparative genome hybridization (CGH), but sometimes also in gene expression and methylation analysis [6]. Chromosome location mapping is processed as follows. Microarray data (log2 ratio between two hybridized genomes) are sorted based on their chromosome location. A quadratic loess curve, which can be viewed as a locally weighted polynomial regression curve through each data set, is constructed. The regions in which contiguous segments of the loess curve were consistently greater than (or less than) a user-defined value times standard deviations away from the mean of the all the data points is identified, and the Mann-Whitney U test is used to determine whether each selected region differed significantly from the set of data points from regions that had not been selected for examination by this test [6].

## Implementation

WebArray runs on a LAMP system (Linux + Apache + MySQL + Python) system. Python was setup with packages: Numeric Python, Rpy, Karrigell and pycrypto. Background computations are mostly done by R scripts. The source code is distributed under the GNU General Public License and is freely available for non-profit use via a request to the authors.

**Figure 1:**
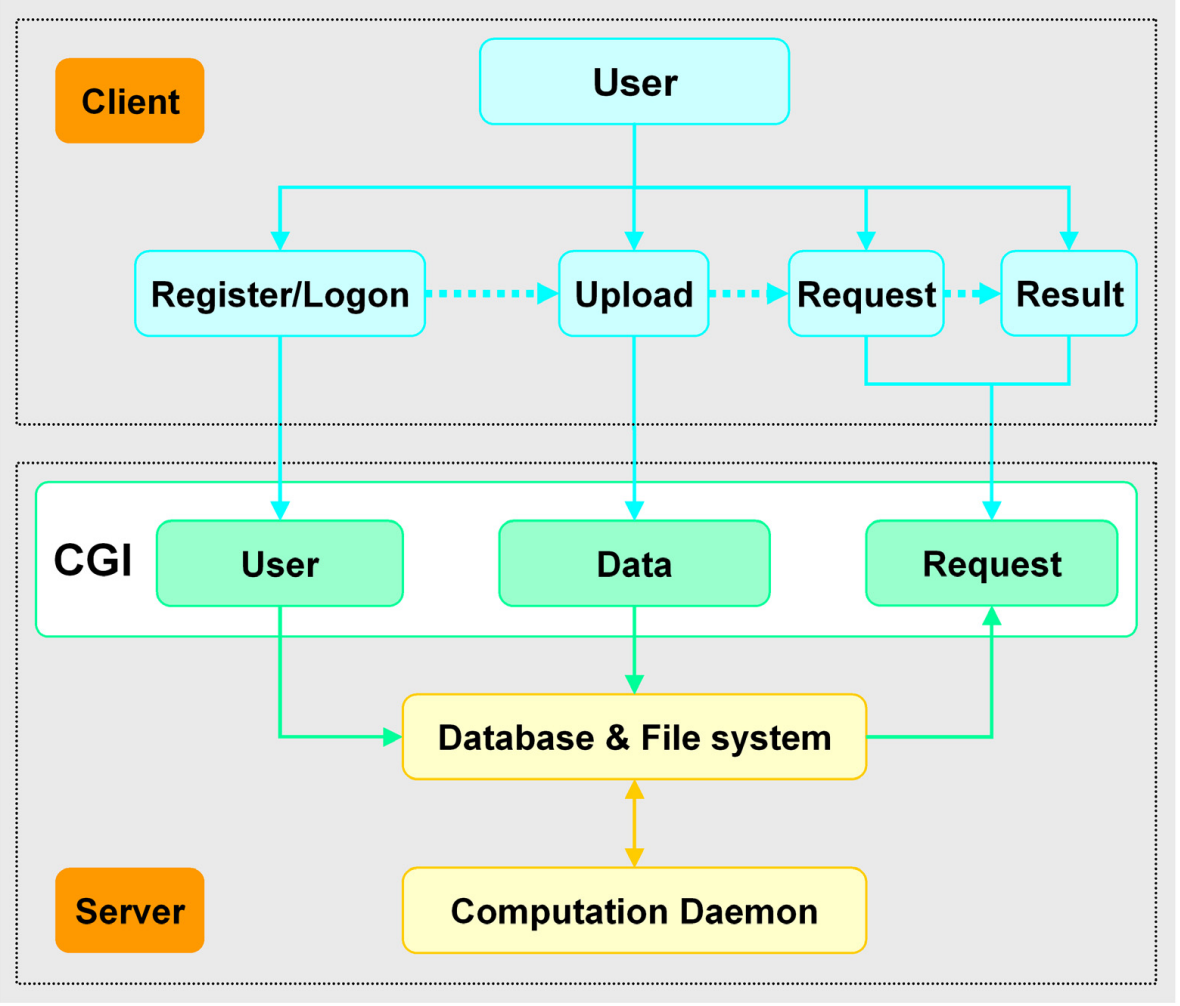
WebArray client-server architecture.

On WebArray server, python CGI scripts deal with communication between users and the server. As shown in Figure 1, the functions of these scripts fall into three categories: 1) user management, including user registration and signing; 2) data management, including uploading data and maintenance; 3) Requests management, i.e., accepting/deleting requests and browsing results. User information, data files, submitted jobs and results are stored in database and server file system. A scheduler program deals with request jobs.

**Figure 2:**
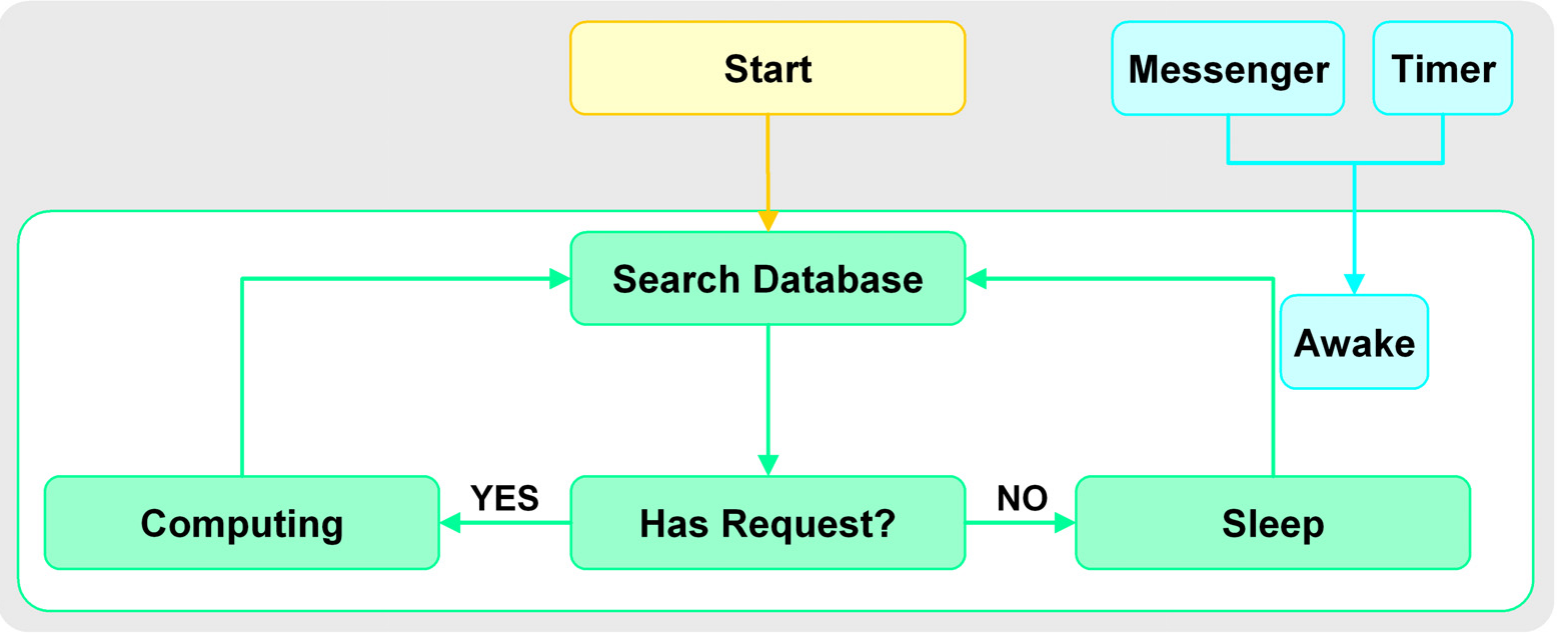
Control logic for WebArray job scheduler.

The scheduler maintains a searching-dealing loop, its scheduling logic is shown in Figure 2. Once launched, this program will check new jobs in the database. New jobs are delivered in turn to the corresponding R script for computing. In case of no new job found, the scheduler will go to sleep. After a request is submitted to the database, CGI will send a message to invoke the scheduler and restart its work cycle. A timer is also set to awake the scheduler regularly, this can make sure that the system will work even if errors occur in communication with CGI.

On the client end, users logically follow a four-step course: register/logon, upload data, submit requests and browse results (see Figure 1). JavaScript is used to enhance the user interface.

## Results and discussion

**Figure 3:**
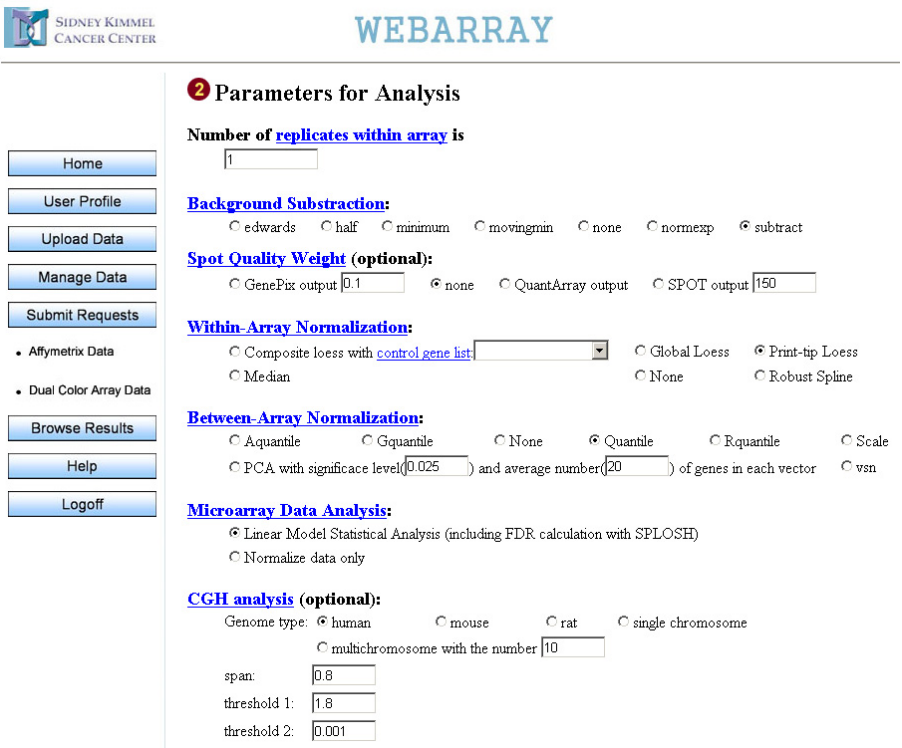
Snapshot of WebArray web page for dual color array data analysis.

Microarray analysis using WebArray can be executed in three steps: 1) uploading and managing files; 2) selecting datasets and methods for analysis; 3) browsing results. Partial web page for dual color array data analysis is shown in Figure 3. A help document is available online with detailed annotation of all functions of WebArray.

As the first step for analysis, users need to upload their microarray intensity files, gene list file and others. The files will be deleted from the server six months after submitting. Users can view and manually delete these files, as well. WebArray requires the following files for analysis; 1) Intensity files. Text files exported by a variety of image analysis programs such as Affymetrix, Agilent, ArrayVision, Genepix, ImaGene, QuantArray, SMD and SPOT. Files exported from other programs have to be uploaded in a specified format; 2) Targets file. A tab-delimited text file listing the targets hybridized to each channel of each array; 3) Gene list file, such as gene allocation list (GAL) file. A specified format is acceptable too; 4) Design file. A tab-delimited text file containing design matrix for linear model; 5) Spot type file (STF). STF is used to distinguish different types of spots from the gene list using regular expression, including control spots, positive and negative controls; 6) Genome/chromosome location file. A tab-delimited text file containing array spots sorted by genome/chromosome location information; 7) control genes file. A text file containing housekeeping gene's printing order index for composite normalization. Intensity files are required for all analysis, a gene list file is required for dual color array data analysis and all other files are optional.

In the "submit requests" page, users can select data for analysis from their own uploaded files. WebArray includes most of the functions limma provided, such as spot quality weight, background subtraction, normalization and empirical Bayes statistical analysis. In addition, principal component analysis assisted normalization method is incorporated [7], FDR can be estimated using SPLOSH, and chromosomal mapping will be plotted if desired.

The limma package uses linear models to analyze designed microarray experiments. For Affymetrix array data and simple dual color experiments, such as two-sample comparison with switching dye or two-sample comparison with common reference, users can specify the design just by selecting sample types in the columns corresponding to each microarray intensity file. For multi-sample comparisons or complicated experiment design, users need more statistical knowledge for the creation of design matrix and contrast matrix.

WebArray allows the user to name a request, otherwise a name will be assigned automatically. Submitted requests will be put on a waiting list. The page for results allows users to browse their own list of requests. Requests can be edited or removed. Since computation with microarray data usually involves huge data sets, it may take a few minutes to complete a computation. For data sets with within-array duplicates the process will take much longer time, maybe hours.

**Figure 4:**
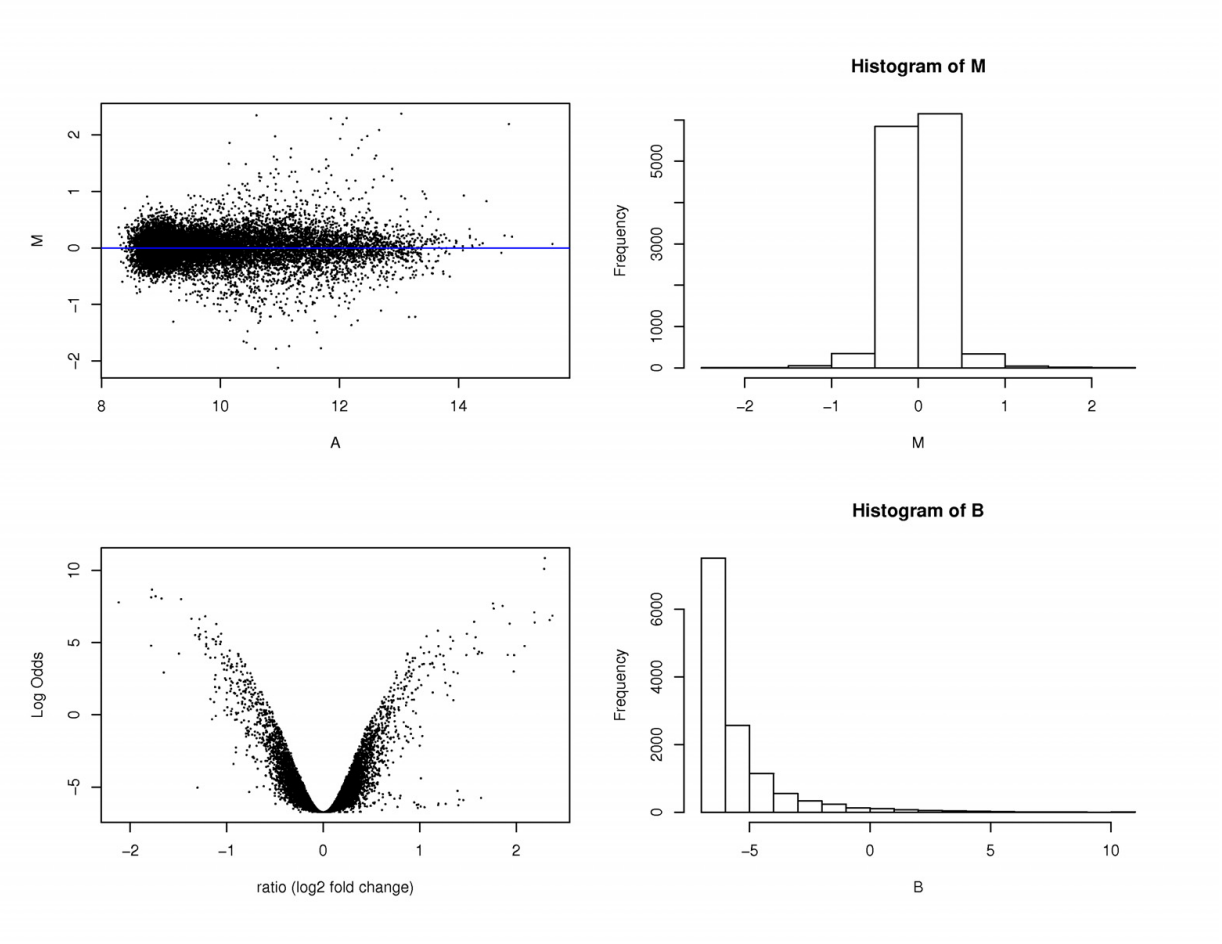
**Statistical analysis result plot. **Result plot includes M-A plot, M-B plot, M histogram and B statistics histogram. M: the log-differential expression ratio. A: the log-intensity of the spot, a measure of overall brightness of the spot. B: B statistics, the log-odds of differential expression.

The output files include tab-delimited text files and graphic plots that can be downloaded separately or viewed online. All the files are archived in one ZIP file and are available for download as well. Based on the options of analysis, the text file may contain the gene information, M (log2 ratio), moderated t and its corresponding p-value, B statistics, FDR, FP (false positive), FN (false negative) and CGH fitted value. The table of genes can be either unranked or ranked by M, p value or B statistics. Graphic plots include array image plots, density plots, histogram, RNA degradation plot, M-A plot for each array (before and after within-array normalization), printtiploess plot for each array, box plot (before and after between-array normalization), chromosomal location mapping plots where M is plotted against chromosomal location and results plot that includes M-A plot, M-B plot, M histogram and B statistics histogram (see Figure 4).

While more sophisticated programs are available commercially, WebArray represents an excellent free open source software for microarray analysis that can be used by an average biologist after moderate training. To help biologists to understand the underlying statistics methods, we provide detailed explanations and references for most WebArray functions in the help document.

## Availability and requirements

• Project name: WebArray

• Project home page: 

• Operating system(s): Platform independent (web-service)

• Programming language: Python, R.

• Other requirements: Internet browser, such as IE, Netscape or Firefox, with cookie and JavaScript support enabled. For the purpose of data management and security, users need to register an ID and password on WebArray prior to use.

• License: under the GNU General Public License [8] for download.

## Authors' contributions

XX performed software engineering, coding and debugging. MM was involved in the guidance of this project. YW designed the website and wrote the R code. All authors read and approved the final version of the manuscript.
